# 

**Published:** 1890-12

**Authors:** 


### LITERARY.

THE HOME MAKER.—An illustrated monthly of 100 pp. Edited by the popular and well known authoress, "Jenny June." We have made arrangements with this excellent magazine to supply it and our JOURNAL OF HEALTH for $1.50 per annum ; less by $1 than the regular subscription price of the *Home Maker.* This is a rare offer and should be largely availed of.

THE COTTAGE HEARTH; Boston. A most excellent monthly periodical, now in its 17th year. We are prepared to offer our JOURNAL, with the above, for $1.50 per annum, the price of the former.

AMERICAN OTOLOGICAL SOCIETY.—Transactions of the 23d annual meeting, July, 1890. This is an illustrated pamphlet of 666 pp., with a very complete index to subjects, and reflects great credit upon the Society and the profession.

REVUE INTERNATIONALE DE BIBLIOGRAPHIE MÉDICALE, PHARMACEUTIQUE ET VÉTÉRINAIRE, Paris and Beyrouth, Syria. Vol. II., No. 3.—This is a complete index to the current medical publications the world over ; invaluable to the medical profession as a reference book.

THE CENTURY MAGAZINE.—This reliable monthly grows in interest with every issue. Its woodcuts are models of an art which has made wonderful strides the past few years, and now stands next in artistic merit to the costly steel line engravings.

LIFE is the title of a new and very interesting book of nearly 300 pages, devoted to a very full and comprehensive discussion of the occult forces as exemplified in Mesmerism, Clairvoyance and the various phases of what are usually termed spirit phenomena. The author, W. W. Wheeler, of Meriden, Connecticut, has evidently mastered the spiritual philosophy and become convinced of its truth. Without any sort of introduction or prefatory explanation of its purposes, the author launches at once into his theme by presenting a mesmeric sensative in a comatose state, whose devoted sister is fighting an unequal battle with the health authorities to prevent his premature interment. The interest is well sustained throughout the work and various forms of occult phenomena presented in a clear and appreciative manner, making the book a valuable addition to this class of literature, may we not add, knowledge. Price, 50c.—*American News Company.*

